# Recent Perspectives on Sex Differences in Compulsion-Like and Binge Alcohol Drinking

**DOI:** 10.3390/ijms22073788

**Published:** 2021-04-06

**Authors:** Anna K. Radke, Elizabeth A. Sneddon, Raizel M. Frasier, Frederic W. Hopf

**Affiliations:** 1 Department of Psychology and Center for Neuroscience and Behavior, Miami University, Oxford, OH 45040, USA; sneddoea@miamioh.edu; 2 Department of Psychiatry, Indiana University School of Medicine, Indianapolis, IN 46202, USA; rmsandle@iu.edu (R.M.F.); whopf@iu.edu (F.W.H.)

**Keywords:** sex differences, alcohol, nucleus accumbens core, nucleus accumbens shell, dopamine, orexin, AMPA (α-amino-3-hydroxy-5-methyl-4-isoxazolepropionic acid), early life stress, anxiety, rodent models

## Abstract

Alcohol use disorder remains a substantial social, health, and economic problem and problem drinking levels in women have been increasing in recent years. Understanding whether and how the underlying mechanisms that drive drinking vary by sex is critical and could provide novel, more targeted therapeutic treatments. Here, we examine recent results from our laboratories and others which we believe provide useful insights into similarities and differences in alcohol drinking patterns across the sexes. Findings for binge intake and aversion-resistant, compulsion-like alcohol drinking are considered, since both are likely significant contributors to alcohol problems in humans. We also describe studies regarding mechanisms that may underlie sex differences in maladaptive alcohol drinking, with some focus on the importance of nucleus accumbens (NAcb) core and shell regions, several receptor types (dopamine, orexin, AMPA-type glutamate), and possible contributions of sex hormones. Finally, we discuss how stressors such as early life stress and anxiety-like states may interact with sex differences to contribute to alcohol drinking. Together, these findings underscore the importance and critical relevance of studying female and male mechanisms for alcohol and co-morbid conditions to gain a true and clinically useful understanding of addiction and neuropsychiatric mechanisms and treatment.

## 1. Introduction

Despite considerable efforts across a number of decades, alcohol use disorder (AUD) continues to be a substantial health problem, with significant economic, social, medical, and legal costs [1,2,3,4,5,6]. Alcohol problems are especially caused by binge drinking, which involves high levels of alcohol intake that reach 80 mg/dL (the legal blood alcohol limit for driving in the U.S.). About 3/4th of the costs related to AUD are due to individuals who binge [6]. In addition, reducing binge consumption can lower health risks [3,5] and the incidence of relapse [1] in dependent drinkers, while binging in non-dependent drinkers can promote development of more significant alcohol problems [7,8,9]. In recent years, particular attention has begun to be focused on so-called High Intensity Drinking, where intake in one session is more than twice the legal limit, with the recognition that the level of harm increases in proportion to the level of binging [10,11].

In addition to binge intake, another important contributor to alcohol problems is compulsion-like alcohol drinking, where intake persists despite negative consequences. The choice to keep drinking even in the face of known harms (such as loss of job, family, or freedom) has become a defining feature of the Diagnostic and Statistical Manual of Mental Disorders (DSM)-5 criterion for AUD [9,12] and is considered a potent obstacle to treating AUD [1,3,4,6,13,14,15,16]. Thus, there has been considerable recent effort to uncover critical underlying mechanisms of both binge and compulsion-like intake. One goal is to identify key mechanisms that drive these pathological forms of alcohol consumption, since these may provide novel and translationally useful therapies to reduce the burden of alcohol-related costs. This effort is considered especially critical since, at present, there are few pharmacotherapeutic options approved for AUD treatment [17].

To facilitate the identification of critical underlying molecular and brain circuit mechanisms associated with alcohol drinking, addiction researchers have widely turned to preclinical animal models. In particular, rodents are a relatively inexpensive model (vs. non-human primates) that allow invasive mechanistic investigation. Indeed, C57BL/6J (C57) mice and selected mouse and rat strains will drink substantial amounts of alcohol, opening the possibility of uncovering central underlying mechanisms that drive the urge to drink alcohol in a binge-like fashion. One critical question is whether the mechanisms that mediate rodent alcohol consumption accurately reflect circuits and signaling systems that promote human intake. Although beyond the scope of this review, a large literature has implicated a number of brain regions in promoting alcohol consumption in rodents, with somewhat comparable circuit findings in humans with AUD [15,18,19,20]. However, limited advancements have been made in identifying new treatments for AUD. While this could be due to a number of factors, including differences in core mechanisms across individuals and the importance of drinking context and related factors (including social pressures that cannot be modeled in humans), the limitations of standard rodent drinking models are a potential factor. Thus, the use of behavioral paradigms that capture key characteristics of AUD may increase the translational impact of rodent models of alcohol drinking.

In addition to studying binge-like drinking patterns in rodents, there is growing interest in aversion-resistant consumption, which has been considered to model some aspects of compulsive, consequence-resistant, drinking motivations in humans (described above). However, it is important to note that while resistance to negative consequences is likely a critical contributor to addiction, it is only one of several potential factors, including greater valuation of rewards and greater reactivity to drug-related cues [21,22]. Further, there are different considerations about whether individuals with AUD present greater insensitivity to cost [23,24,25] or not [21,26] (reviewed in [27,28]). There are also multiple ways to conceptualize what compulsion reflects, from a stronger form of habit to a goal-directed process of overcoming conflict (which likely recruits habit as well as other circuits) (reviewed in [27,29]). Another critical consideration is the timing of the adverse consequence relative to intake, where negative outcomes often occur in the future for humans (e.g., loss of job, legal problems), while rodent models involve acute punishment or adversity. Threading this needle, we consider findings from our rodent compulsion-like intake models to better reflect mechanisms active in treatment-seeking humans or in people who recognize their drinking problems but are not yet in treatment. In particular, it is likely that treatment seekers are actively struggling with the balance between known bad outcomes and the desire to drink, meaning that the possibility of negative outcomes are much more acute. Compulsion-like drives are somewhat difficult to study in humans, although both DSM-5 and the Alcohol Use Disorders Identification Test (AUDIT), another commonly-used method to assess addiction level, contain multiple questions which indicate self-report of problems associated with drinking. Nonetheless, given the central importance of consequence resistance in DSM-5 and AUDIT, and their greater prevalence in High Intensity Drinking [10,11], this is an area of great importance that requires considerable additional attention. Thus, even if rodent findings are only relevant to a smaller range of human addictions, their ability to uncover mechanisms relevant to treatment seekers continues to inspire rodent investigators.

In the quest to uncover mechanisms underlying binge-like and compulsion-like alcohol drinking and to increase the translational impact of such studies, one critical consideration is the inclusion of both male and female subjects in study designs. While the field of neuroscience has historically, and unjustifiably, excluded female subjects from the majority of studies [30], clinical work suggests a worrying vulnerability to alcohol in female patients. Women are more likely than men to experience health problems caused by their drinking [31,32] and may become dependent faster [33,34,35]. Of particular concern is the fact that rates of binge-drinking and AUD in the United States have been increasing in women, while remaining stable or declining in men [36,37,38,39,40]. Such trends highlight the need for a clearer understanding of the effects of alcohol on the female brain and female-specific mechanisms involved in dependence. Here we review recent findings from our labs and others regarding female vulnerability to binge and compulsion-like alcohol drinking. We find that female rodents have a heightened propensity for binge and compulsion-like behaviors, which is associated with sex-specific mechanisms in the nucleus accumbens (NAcb).

## 2. Female Vulnerability to Binge and Compulsion-Like Alcohol Drinking

One often-used approach to assess binge-like alcohol consumption in rats [41,42] and mice [43,44,45,46] is a limited access paradigm known as drinking in the dark (DID) [47]. In this procedure, rodents are given access to a single bottle of alcohol starting three hours into the dark cycle and drink for up to 4 h [47]. Compared to many other home-cage alcohol-access paradigms, DID results in pharmacologically significant blood alcohol concentrations (BAC) that reach binge level (>80 mg/dL) and correlate with g/kg intake in both sexes (though it should be noted that higher average intake in females does not always produce higher average BACs) [45,48,49,50]. Although widely employed, many early DID studies used only male rodents [41,46,51,52,53,54,55,56,57,58,59,60,61,62,63,64] or collapsed the data by sex [50,63,64,65]. The Radke lab examined binge-like drinking of 15% alcohol in male and female C57 mice in a two-bottle choice version of the DID paradigm and, consistent with earlier work using this line of mice [44,49,50,66], found that females consumed significantly more alcohol than males (>1 g/kg more than males) [43]. Interestingly, in our study, higher levels of consumption in females did not emerge until after the 10th exposure session, suggesting an influence of exposure history on the development of this escalated pattern of drinking in females; such escalation has been seen in some intermittent-access drinking schedules [67] but not others [68]. Greater female intake has also been observed in mouse lines selected for high DID behavior (HDID-1 and HDID-2 mice) [45].

Collectively, these results suggest that rodent models of binge drinking, like DID, are particularly useful in capturing a female tendency to greater alcohol self-administration. Greater consumption in female vs. male rodents in DID is observed across alcohol concentrations and experimental variations such as length of the exposure session and presence or absence of concurrent access to water during drinking sessions. Higher consumption by female vs. male rodents has also been reported in 24-h continuous [67,69,70,71] or intermittent-access paradigms [67,72,73,74,75] and in operant response tasks [76], although other studies using these methods have not seen sex differences in intake [77,78,79,80,81]. In addition to the tendency to induce binge-like intake patterns, DID paradigms may consistently engender a sex difference in consumption levels because higher concentrations of alcohol are typically offered. Indeed, we (Radke lab) recently demonstrated that female C57 mice self-administer more 15% alcohol on a fixed ratio (FR) three operant response schedule than males, but demonstrate no differences when 10% alcohol is available [76]. Similarly, Wistar rats with continuous access to alcohol show concentration-dependent sex differences in consumption, with greater female intake only when alcohol was greater than 8% [69].

Considering the tendency of females to exhibit more binge-like alcohol drinking than males in DID and other alcohol access paradigms, it is not surprising that female vulnerability to compulsion-like drinking has also been observed. In rodent models, compulsive behavior can be modeled by pairing delivery or ingestion of alcohol with acute punishment [15,82,83,84]. Foot-shock and aversive tastants such as quinine are the two punishments most commonly used and, despite differences in the neural circuits activated by each of these aversive stimuli, similar neural mechanisms have been found to underlie alcohol drinking in male rodents when punished with quinine vs. foot-shock [18,85,86]. The Radke lab has observed greater resistance to aversion/punishment in female rats [80] and mice [43,76]. Using an intermittent access home cage drinking paradigm, we found that female Long Evans rats tolerated higher concentrations of quinine in 20% alcohol during 30-min drinking sessions than male rats [80]. In the two-bottle choice DID task when quinine is added to the alcohol bottle from the first drinking session, female C57 mice escalate their intake and consume more quinine-adulterated alcohol than males [43]. We have also demonstrated that, under operant conditions (responding for 0.5 μL of 10% alcohol on an FR3 schedule of reinforcement), female mice continue responding for alcohol adulterated with higher concentrations of quinine than males [76]. In addition, female C57 mice will continue to consume 15% alcohol (in a continuous access paradigm) at higher concentrations of quinine relative to males [87], and female mice show little reduction in alcohol conditioned place preference (CPP) when the alcohol-paired chamber is paired with foot-shocks, while shock-pairing does reduce male CPP [88].

Although we have not found reports of greater compulsion-like drinking in male rodents, it is clear that not all experimental designs show greater female compulsivity for alcohol [89]. For example, when C57 mice first escalate intake of quinine-free alcohol across twelve sessions of two-bottle choice DID, addition of quinine to the alcohol bottle produces similar decreases in consumption across sexes [43]. This result has been replicated in C57 mice consuming 20% alcohol in a one-bottle variant of the DID procedure [90]. Further, in a chronic, continuous three-bottle choice paradigm (20% alcohol, nicotine, and water), male and female mice similarly reduced consumption when quinine was added to the alcohol [91]. An absence of sex differences in quinine’s ability to reduce breakpoints for alcohol under a progressive ratio (PR) schedule in rats has also been reported [89]. We also note that while both alcohol exposure history and a history of early life stress can increase compulsion-like alcohol drinking, we have not found female vulnerability to compulsion to interact with either of those variables [80,92]. It is noteworthy that DID, a paradigm that reveals robust binge drinking, appears to mask sex differences in compulsion-like drinking [43]. One possible explanation is that higher levels of consumption or rapid intake under DID may lead to greater, desired intoxication, while models with lower or slower intake (e.g., slower under operant, lower alcohol concentration, less front-loading of intake under continuous access) may be associated with greater motivation to consume despite punishment in females vs. males. While these ideas remain to be tested, differences among paradigms used to test compulsion-like alcohol drinking in rodents highlight the importance of considering the behavioral parameters of the study design when assessing potential sex differences in alcohol-drinking behaviors.

Inherent to the design of compulsion models is the balance between the appetitive drive for the drug and avoidance of the aversive stimulus. It is increasingly clear that these two opposing drives can be mediated both by some common circuitry (e.g., where signaling indicates salience rather than positive or negative valence) and also distinct pathways. Thus, increased expression of compulsion-like drinking in females (vs. males) could result from increased appetitive motivation for alcohol, decreased sensitivity to aversion/punishment, or both. We have repeatedly demonstrated that male and female rodents equally avoid quinine solutions in water, both before and after alcohol exposure [43,76,80], and find no differences in primary shock sensitivity (using the flinch-jump-vocalization test) between male and female C57 mice [88] (Sneddon et al., unpublished data). These results suggest that sex differences in compulsivity under many conditions reflects more than basal differences in aversion sensitivity between male and female animals.

## 3. Ovarian Hormones Promote Binge Alcohol Drinking

Estrogens are known to have a facilitatory influence on some reward-seeking behaviors, an effect mediated at least in part by actions on striatal dopamine signaling [93,94,95]. In addition to the important role that sex hormones play during brain development (i.e., organizational influences) [96], there are two ways in which circulating hormones such as estrogen can have ongoing (activational) influences on alcohol drinking and other behaviors. The first is through the daily fluctuations associated with the rodent estrous cycle, which consists of four stages (proestrus, estrus, metestrus, and diestrus) and cycles every four to five days in mice [97] and every four days in rats [98]. Examinations of estrous cycle effects on behavior are best accomplished by daily karyotyping of cells obtained by vaginal lavage. Higher levels of estrogens in females vs. males may also impact behavior independent of cycle-related variations in hormone levels. As such, another method to explore estrogen effects on alcohol drinking behaviors is to ovariectomize (OVX) a female rodent, then examine the impact of restoring exogenous estradiol, which allows control over hormone levels but lacks the cyclicity of intact females.

In regards to variations in estrogens across the cycle, the majority of studies report no influence of estrous cycle phase in intact females on consumption or preference [44,73,74,75,87,99]. Binge alcohol consumption does not vary with estrous cycle phase in mice drinking 20% alcohol for 4 h in the dark [44]. Another study examining compulsion-like drinking of continuously-presented quinine-adulterated 15% alcohol also found no influence of estrous cycle phase [87]. When alcohol drinking behaviors are influenced by the estrous cycle [81,100] the effects tend to be modest, for example manifesting as changes in drinking patterns across phases but not changes in total intake [101] or occurring only in rats with adolescent alcohol exposure [102]. Despite this relative lack of estrous cycle effects in alcohol drinking, there is physiological evidence suggesting that excitation of ventral tegmental area (VTA) dopamine neurons by alcohol is greatest in diestrus, when circulating estradiol levels are high [103]. Alcohol-induced dopamine release in the prefrontal cortex has also been shown to be dependent on cycle phase [104]. Thus, it is possible that estrous cycle phase impacts sensitivity to alcohol in ways that are not easily captured with blunt measures like total consumption, and more precise behavioral approaches such as operant response paradigms and examination of licking microstructure (e.g., to examine the rate of intake, as an indicator of motivation) could provide important translational insights lacking in more general measures. Further, across studies of alcohol and co-morbid conditions such as anxiety disorders, even though there can be mixed results, there are certainly conditions under which estrous cycle phase can strongly impact behavioral expression [74,105,106]. Thus, this continues to be an important area for future investigation.

Although estrous cycle phase appears to have limited effects on alcohol drinking behaviors, studies using OVX have presented somewhat different results. For example, in one study, higher binge drinking in female mice was reduced to male levels following ovariectomy and female-typical drinking levels were restored with exogenous estradiol treatment [44]. Other OVX studies suggest that estrogen can facilitate drinking in models of moderate alcohol consumption such as continuous home-cage access [107,108,109,110,111,112] and operant self-administration [113]. There remain some mixed findings on the impact of ovariectomy and/or hormone replacement [69,114,115,116] and it is unclear how much these inconsistencies relate to differences in drinking model, species, strain, and perhaps other factors. On the whole, it appears that the average higher levels of circulating estrogens in females vs. males can contribute to sex differences in drinking behaviors more so than cyclical fluctuations in estrogen levels in females. Evidence that estrogen potentiates alcohol-induced excitation of neurons in VTA [103] and that targeted knockdown of estrogen receptors in the VTA reduces binge alcohol drinking in female, but not male, mice [117] suggests a role for increased activity in the mesolimbic dopamine pathway in estrogen-mediated facilitation of drinking in female animals.

## 4. NAcb Core in Binge and Compulsion-Like Alcohol Drinking

The NAcb is a critical node in the circuits mediating motivated behaviors directed by both appetitive and aversive stimuli [118,119]. Both subregions of the NAcb, the core and shell, are involved in alcohol drinking behaviors. For example, alcohol induces synaptic changes in the NAcb core, and this subregion is known to promote alcohol-seeking and escalation of intake [53,120,121,122,123,124,125,126,127,128,129]. Likewise, manipulations of NAcb shell also reduce alcohol consumption [130,131,132] (see below). The NAcb is a sexually dimorphic region [133,134] where females display greater dendritic spine density, larger dendritic spine heads, and greater variability in spine density than males [135,136]. Further, there are also known sex differences in NAcb physiology, including facilitated dopamine release and neuronal excitability by estradiol [137,138,139,140], that are likely to contribute to enhanced reward-seeking behavior in females vs. males. As such, the NAcb is of considerable interest for our studies of binge and compulsion-like alcohol drinking.

The conceptualization of the NAcb core providing a go/no signal for goal-directed behavior, as well as mediating the vigor of responding [118], suggest that this region could act through several different mechanisms to promote excessive alcohol drinking in binge and compulsion-like drinking paradigms. Indeed, manipulations of NAcb core modulate binge drinking in DID paradigms and sex-dependent effects of such manipulation have been observed [132,141,142]. For example, recent studies using chemogenetic technology to inhibit the NAcb core in C57 mice drinking 20% alcohol using DID have reported reduced consumption in males but not in females [142,143]. These studies also found evidence for reduced consumption following chemogenetic excitation, but in this case in females and not males [142,143].

The Radke lab recently evaluated the contribution of NAcb core medium spiny neuron (MSN) subtypes and dopamine receptors (D_1_-like and D_2_-like receptors) to binge and compulsion-like alcohol drinking using two-bottle choice DID in male and female C57 mice [144]. We found that binge alcohol drinking (15%) on quinine-free sessions was not affected by selective chemogenetic inhibition of D_1_-receptor expressing “direct” pathway neurons (D_1_-neurons) or D_2_-receptor expressing “indirect” pathway neurons (D_2_-neurons). However, using pharmacology, we found that combined systemic antagonism of D_1_ and D_2_ receptors reduced alcohol consumption in male, but not female, mice. This result suggests that NAcb core dopamine signaling may regulate alcohol drinking in a sex-dependent manner, with importance for male but not female mice (which, interestingly, mirrors what is seen with NAcb shell orexin-1 receptors, described below).

We next looked at regulation of quinine-adulterated DID alcohol intake by NAcb core cells [144]. Here, chemogenetic inhibition of NAcb core neurons (i.e., without targeting a particular cell type) reduced compulsion-like drinking similarly in females and males, while targeted inhibition of D_1_-neurons or D_2_-neurons was ineffective (similar to alcohol-only DID intake above). Further, combined, but not separate, antagonism of D_1_ and D_2_ receptors reduced compulsion-like alcohol drinking in a similar manner, again with similar effects in females and males. Together, our results show that inhibiting NAcb core D_1_-neurons alone or D_2_-neurons alone is not sufficient to alter binge or compulsion-like drinking in either sex, while inhibiting NAcb core neurons more generally reduced compulsion-like intake similarly in females and males (not tested for binge intake). Further, combined D_1_R/D_2_R inhibition reduced male but not female binge drinking, and reduced compulsion-like intake in both sexes. Our studies also add to the previous literature implicating NAcb core in punished alcohol drinking behaviors in rats [18,145,146] and support a lack of sex differences in the expression of compulsion-like drinking using this DID paradigm [43,90]. Future studies into mechanisms underlying potential female vulnerability to compulsion-like drinking should utilize intermittent home-cage access or operant response paradigms, where basal sex differences in expression of compulsivity are observed [76,80].

## 5. Sex Differences in NAcb Orexin and AMPA Receptor Regulation of Binge Alcohol Drinking

As described above, the NAcb is considered critical for regulating many motivation-directed behaviors including different forms of alcohol drinking. The NAcb is also involved in stress-driven responding, such as early life stress (discussed below) and compulsion-like responding (where responding persists in the face of an aversive stressor, see Introduction). Thus, it is of great interest to understand whether sex differences in NAcb signaling could promote sex differences in alcohol consumption. In this section we will review a series of studies by the Hopf lab, which focus on the NAcb shell subregion. The NAcb shell mediates many motivated and addiction-related behaviors [119], including cue and context reinstatement for alcohol [147,148] and alcohol intake under punishment [149], and other compulsion-like behaviors [150,151] including habitual resistance to devaluation of reward [151], which is conceptually similar to compulsion-like responding. Many studies have also examined the NAcb shell’s importance for alcohol intake, with multiple groups concurring that inhibition of NAcb shell or NAcb more generally reduces alcohol drinking, without inhibiting control behaviors such as intake of sweetened fluid and locomotion [130,132,152,153,154,155,156,157].

### 5.1. NAcb Shell Orexin Regulation of Alcohol Drinking in Male Mice

The Hopf lab is particularly interested in NAcb shell orexin-1-receptors (Ox1Rs) and glutamatergic α-amino-3-hydroxy-5-methyl-4-isoxazolepropionic acid receptors (AMPARs) (which we reviewed in [27,119]). We first will address orexin/hypocretins, which can regulate a wide variety of adaptive behavioral and physiological responses ranging from sleep and food intake to addiction and stress [27,158,159]. Ox1Rs are of particular interest, as many studies converge around the importance of Ox1Rs for driving behavior aimed at procuring high-value natural and drug rewards, including where higher levels of cognitive or physical effort are required; in contrast, Ox1Rs generally have little contribution to behaviors requiring lower effort or when less-motivating substances are delivered [158,159,160,161,162,163]. As an example, cocaine delivery after a single lever press does not require Ox1Rs, but five lever presses for cocaine or an ever-increasing number of lever presses (PR) do require Ox1Rs (see [27]). In addition, systemic Ox1R antagonism decreases pathological forms of drinking, including intake in genetically-alcohol-preferring animals [164] and after induction of alcohol dependence [165] (see [27], and discussed further below). However, Ox1Rs do not regulate alcohol place preference [166], suggesting a role in motivation rather than reward per se [158,159].

Thus, given the importance of the NAcb shell and Ox1Rs (tested with systemic inhibition) for alcohol drinking, we (Hopf lab) have engaged in several studies of NAcb shell Ox1Rs for binge-level alcohol drinking in male and female C57 mice. We will first address our original work in male drinkers, then contrast drinking mechanisms in females vs. males. We first found that inhibiting NAcb shell Ox1Rs (with SB-334867, SB), but not Ox2Rs, reduced alcohol consumption in male mice in a modified DID protocol (2 h/day, 5 d/wk intake, starting 2.5–3 h into the dark cycle, with two bottles, 15% alcohol or water) [152]. Note that this two-bottle version of the DID paradigm, which has also been used by the Radke lab to test binge and compulsion-like drinking (described above), retains intake during the dark cycle but differs from the single alcohol bottle offered in classic DID models [50] (also addressed further below). We also used patch clamp electrophysiology in the NAcb shell, and found that orexin increases evoked firing of NAcb shell neurons through Ox1Rs, although with no differences between controls and alcohol-drinkers. Finally, NAcb shell Ox1Rs do not alter saccharin intake (with saccharin level titrated to give approximately equal volume of intake as alcohol) [152] and NAcb core Ox1Rs do not regulate male alcohol intake [167]. Together, our studies identify Ox1Rs, but not Ox2Rs, in the NAcb shell as important for regulating alcohol intake (in a substance- and region-specific manner) in male mice, potentially through Ox1R activation of NAcb shell firing.

In subsequent studies, we (Hopf lab) showed that NAcb shell Ox1Rs also regulate male binge alcohol drinking under an intermittent access model (three overnights per week, with one day between drinking days) [167]. In addition, global inhibition of the NAcb shell with muscimol/baclofen (an often-used cocktail of GABA receptor activators) depresses male mouse drinking under both DID and intermittent access, consistent with NAcb shell neuron activation (including through Ox1Rs) driving binging. With this very large data set, we combined results across models and inhibitors and found that Ox1R antagonism or more global neuronal inhibition within the NAcb shell have an interesting impact in relation to basal drinking levels. In particular, animals on the lower range of basal alcohol intake (defined as moderate bingers) are on average not impacted by NAcb shell Ox1R or global inhibition. In contrast, drinking levels in higher basal-drinking male mice (defined as excessive bingers) are strongly reduced by NAcb shell Ox1R or global inhibition. Interestingly, previous studies with systemic Ox1R inhibition find a similar pattern, with reduced intake only in higher-drinking rats or mice [168,169,170] but no effect in lower drinkers. Thus, our findings are important in that they identify the NAcb shell as a critical region where Ox1R enhancement of neuronal activity specifically promotes drinking in higher-binging individuals. Effects observed only in excessive bingers could result from a floor effect, so that moderate binging could not be reduced. To assess this possibility, we tested systemic administration of baclofen, a GABAB agonist that has shown promise against alcohol drinking in some human studies [171]. Indeed, baclofen dose-dependently reduced alcohol drinking in all animals, regardless of basal drinking levels, which likely occurs at a site other than the NAcb shell. Thus, our results support the use of baclofen across all drinking levels, while Ox1Rs would be particularly valuable against intake in the most costly, heavy-drinking males. As described also in the Introduction, the majority of the very high alcohol-related costs from human drinking are extracted by heavier-drinking bingers. Thus, Ox1Rs may be particularly valuable therapeutically in reducing consumption in the critical target population of heavier bingers, and it would be of great interest to use fMRI to determine whether such reduced intake (and perhaps drive to drink) by Ox1R blockers scaled with changes in NAcb activity in heavy-drinking humans.

### 5.2. NAcb Shell AMPAR but Not Orexin Receptors Regulate Alcohol Drinking in Female Mice

Having discovered that NAcb shell Ox1Rs are particularly important for alcohol intake in excessive-binging males, we examined potential NAcb shell Ox1R regulation of female mouse drinking. In particular, since females consume higher levels of alcohol than males in this DID paradigm (described above), we predicted that NAcb shell Ox1Rs are imperative for female drinkers. Surprisingly, we found that inhibiting NAcb shell Ox1Rs had no effect on female mouse alcohol drinking [172], in strong contrast to males. Thus, some aspects of alcohol intake regulation, especially Ox1Rs, seem to play different roles in males and females, addressed further below.

Although NAcb shell Ox1Rs regulated binge drinking in male but not female mice, other types of signaling within the NAcb shell could contribute to female (and male) alcohol consumption. In particular, a specific type of AMPA receptor, the calcium-permeable AMPARs (CP-AMPARs), has been widely studied, since these CP-AMPARs appear in the NAcb shell (and other brain regions) after intoxicant exposure and promote maladaptive behaviors (reviewed in [119]). CP-AMPARs in the NAcb shell are particularly interesting, because their expression is induced in the NAcb shell by a variety of intoxicants and stress-related conditions [119], and several studies show [173,174,175] and suggest [176,177] that alcohol exposure leads to CP-AMPAR appearance in the NAcb shell in male mice. However, published work thus far had not directly tested the importance of NAcb shell CP-AMPARs for alcohol consumption. Thus, we recently [172] used a widely utilized inhibitor of CP-AMPARs, NASPM, to test the importance of NAcb shell CP-AMPARs for alcohol binge intake in male and female C57 mice. Interestingly, and very different from NAcb shell Ox1Rs, inhibiting NAcb shell CP-AMPARs strongly reduced alcohol drinking in both female and male mice, and across all subjects (i.e., not in relation to basal intake levels). Importantly, NAcb shell CP-AMPAR inhibition had no significant effect on concurrent water or saccharin drinking in either females or males. Thus, our results strongly suggest that the NAcb shell is indeed critical for driving binge alcohol drinking in both sexes, through CP-AMPARs, and this promotion of intake is substance specific (no effect on sweet fluid intake). However, NAcb shell Ox1Rs play a very different role, promoting alcohol consumption only in males that are excessive bingers, but not females or moderate-drinking males. We did not perform in vitro patch clamp or biochemical studies to demonstrate shifts in AMPAR composition in female NAcb shell neurons, which remains important for future work.

In previous studies, high levels of systemic Ox1R inhibitors reduce single-bottle DID alcohol drinking in female C57 mice [164]. Thus, to better understand possible Ox1R regulation of male and female alcohol consumption, we examined the impact of systemic Ox1R inhibition. High systemic SB (30 mg/kg, as in [164]) similarly reduces binge alcohol drinking in both sexes [172]. There have been some concerns about the specificity of this dose [27,178,179], although it does not alter concurrent water intake in females or males. Thus, Ox1Rs can regulate alcohol binging in females, although our studies indicate that this effect of systemic inhibition did not occur through the NAcb shell. We did not test lower doses of systemic SB, since Anderson et al. [164] showed that 10 mg/kg does not impact intake in females, and we previously found no effect of this dose in males [52]. We also note that studies have implicated Ox1Rs in other brain regions, including the mPFC [152], amygdala, and VTA [180], in male alcohol binging. We are unaware of similar comparisons in females, and it is clearly of great interest to understand the location where central Ox1Rs regulate female intake.

### 5.3. Systemic Test of Orexin Receptor Regulation of Binge and Compulsion-Like Alcohol Drinking

In addition to binge-like intake, the Hopf lab is highly interested in aversion-resistant, compulsion-like alcohol drinking, since this is likely to be a major contributor to human alcohol problems (see Introduction). Thus, we compared quinine-resistant alcohol intake in females and male C57s. We previously found that compulsion-like drinking in male mice is significantly reduced by a lower systemic dose of SB, 3 mg/kg [52], which is ineffective under many conditions including alcohol-only intake in females [164] and males [52]. Interestingly, systemic 3 mg/kg SB significantly and similarly reduced compulsion-like intake in females and males (without altering concurrent water drinking), and our large data set revealed that SB reduction of quinine-resistance did not correlate with basal drinking levels, suggesting Ox1R contributions across all individuals [172]. While we are only just beginning to identify brain regions where Ox1Rs regulate aversion-resistant intake, these findings do provide compelling evidence that compulsion-like drinking in both sexes is impacted by lower dose Ox1R inhibition, perhaps suggesting a similar circuit mechanism across sexes. Indeed, across the literature, lower SB doses seem to suppress behavior involving other putative high-motivation states, including nicotine intake [181] and alcohol drinking in genetically-preferring rats [164]. Additionally, the NAcb shell [150,182] and Ox1Rs [183] regulate several types of compulsion-like behaviors, and the former is also a region where lower dose receptor inhibition can impact higher-effort responding (progressive ratio), while higher antagonist doses are required to reduce lower-effort responding [184,185]. Thus, our interest in the NAcb shell as a regulator of compulsion-like drinking in both sexes remains.

Together, our results indicate that the NAcb shell (through CP-AMPARs) is critical for driving alcohol binging in both female and male mice, while NAcb shell Ox1Rs only mediate alcohol binging in higher-drinking males; in contrast, low level systemic inhibition of Ox1Rs similarly suppressed compulsion-like intake in males and females. Other findings may provide an interesting and perhaps unifying perspective on sex differences in Ox1R recruitment for different motivated behaviors. In particular, males and females exhibit relatively similar reinstatement for cocaine and sucrose, but the role of Ox1Rs is quite different. Systemic Ox1R inhibitors reduce cue- and stress-related reinstatement in males while affecting only stress reinstatement in females [186,187]. One possibility is that female Ox1Rs are especially important and recruited during more stress-related behaviors, including stress-induced reinstatement (others work) and compulsion-like intake (our work), with female Ox1Rs having less role in more basic-level responding (binging and cued reinstatement, or at least where stress is less likely involved). In contrast, male Ox1Rs would contribute to a broader range of motivated behaviors. Further, Ox1Rs promote operant alcohol responding in male alcohol-preferring P-rats [188] with less of an effect in females [164]. Also, Ox1R inhibition in outbred rats reduces alcohol seeking in males (i.e., responding in the absence of alcohol) [159], but only impacts responding in females if alcohol is present [189]. The mechanistic nature of such differences remains unclear: sex differences in orexin receptors are reported in hypothalamus but not in some cortical and subcortical areas, with some other sex-specific patterns (reviewed in [27]). Thus, there are many potential mechanisms that could contribute to sex differences in Ox1R utilization during behavior.

### 5.4. General Consideration of Possible Differential Orexin Receptor Contributions in Females and Males

While Ox1Rs may contribute differently across female and male motivated behaviors, it is important to note that the details of particular response paradigms could have important and perhaps unrecognized influences. For example, since Ox1R contributions to male binge drinking seem to scale with intake level, we also examined NAcb shell Ox1Rs during drinking under a single-bottle DID paradigm, where drinking levels are greater than under two-bottle [52]. Surprisingly, we found [190] that NAcb shell Ox1Rs do not regulate single-bottle DID intake in males. Perhaps Ox1Rs are important when there are choices, and thus a second cohort initially drank under single-bottle, then later a second, water-containing bottle was added, but NAcb shell Ox1R inhibition still had no effect. Thus, unlike the importance of NAcb shell Ox1Rs for male alcohol binging in DID and intermittent-access two-bottle-choice models, NAcb shell Ox1Rs do not seem to contribute when animals initially drink with a single bottle. Perhaps in agreement, one study found that NAcb shell inhibition did not alter single-bottle alcohol intake in female C57 mice, while NAcb core inhibition actually increased drinking [142]. References in Lei et al. [190] discuss limited but interesting studies suggesting that different mechanisms may contribute under no-choice vs. choice conditions, including single- versus two-bottle access for alcohol. However, adding an alternate choice did not itself lead to NAcb shell Ox1R recruitment, suggesting a more speculative possibility that conditions under which a given behavioral response is learned could “lock in” circuitry needed to perform that response in the future. For example, brain circuits recruited when developing a place preference vary depending on whether there is a choice or not [191]. This is perhaps a cautionary example that, when comparing mechanisms of responding across tasks, details may matter more than might be initially considered.

## 6. Sex Differences in Anxiety Are Likely Important for Alcohol Drinking

As noted in the Introduction, high-risk alcohol drinking and AUD has sharply increased in women, creating a need to parse potential sex differences in mechanisms that drive problematic alcohol consumption. Interestingly, women also have a higher prevalence of pathological anxiety and depression, with nearly twice the risk of developing such conditions than men [192,193]. In addition, anxiety-related states have long been considered an important contributor to problematic alcohol intake [194,195,196], perhaps due to the anxiolytic effects of drinking. Therefore, sex differences in mechanisms of anxiety-like responses are likely to be central for alcohol misuse.

At present it is unclear why these sex differences exist, although both innate-biological and socio-cultural mechanisms likely drive such pathologies in humans. In this regard, rodent models can provide much-needed insight by disentangling the contribution of biological mechanisms to these differences. One challenge is that, historically, rodent models of anxiety were developed and validated in males alone, and, while several widely used anxiety-related rodent models have been tested in females, the basic interpretations of behaviorally-based changes may be different across the sexes [194,195,197,198]. The research addressing female anxiety-like responses is somewhat mixed. For example, while female rodent anxiety-like behavior is lower than males in an elevated plus maze and open field (see [199]), other studies find greater female anxiety-like behavior with predator odor (see [200]) and foot-shock induced licking [201,202]. Additionally, females generally display greater locomotion than males during open field tests and other anxiety tasks [203,204,205], and seem to show greater active responses under several anxiety-related conditions, including where females tend to dart (whereas males tend to freeze) in response to fear [197], females avoid rather than freeze in a shuttle-box task [203], and females display more active defensive behaviors under some social stressors vs. males [193,200,203]. Speculatively, female behavioral patterns may reflect a greater need to defend against the presence of danger near a nest [193,194,206]. Thus, studies of sex differences in male and female anxiety have some interesting diversity of behavioral patterns and are in need of greater depth of consideration and study.

We note that, beyond sex differences, there is a larger debate over the validity of rodent measures of anxiety-like behavior. Many promising pharmacotherapies for anxiety in rodent models have failed in human clinical trials [207,208], leading to substantial reconsideration of the use of rodent models. For example, the widely-used elevated plus maze task has not held up under scrutiny as a reasonable analog to human mental disorders [209,210]. In addition, behavioral interventions for anxiety such as forms of talk-therapy cannot be realistically modeled in rodents [210]. Adding to these challenges, anxiety-related conditions in humans are not a clear-cut singular process with one or two discrete behavioral outcomes, but are instead multi-faceted and multi-symptomatic [211]. Indeed, the recently developed Research Domain Criterion (RDoC) from the National Institutes of Health (NIH) was designed in part to help develop new paradigms to address substantial challenges to and gaps in our understanding of neuropsychiatric mechanisms in humans (separate from the additional limitations in putative rodent models of clinical states). Within this new framework, there is a strong push for rodent scientists to reformulate anxiety models: rather than seek face validity for “human anxiety,” instead to capture measurable cognitive and emotional features that mirror those presently defined in RDoC. As an example, the elevated plus maze likely involves many factors including unconditioned emotional responses, decision making, and perhaps some short-term learning, and model improvement to better disambiguate these different factors is a central requirement of the RDoC strategy.

While much remains to be studied regarding sex differences in anxiety contributions to alcohol consumption, the Radke lab has identified effects of early life stress on compulsion-like alcohol drinking that parallel changes in learned acute threat (fear) behaviors (i.e., freezing in response to anticipated shock). The stress-enhanced fear learning (SEFL) model of early life stress-exposure uses a series of foot shocks to mimic a traumatic experience in infancy (PND 17) [212,213]. Rats and mice exposed to PND 17 foot shock exhibit greater fear learning and resistance to fear extinction as adults [212,214], and we found that rats exposed to PND 17 foot shock consumed higher concentrations of quinine in alcohol, regardless of sex [80]. Considering the important contribution of stress and anxiety to pathological alcohol consumption, the effects of infant trauma on learned fear and compulsion-like drinking are likely to depend on shared mechanisms. Further, our unpublished preliminary data demonstrate a female vulnerability to the effects of infant foot shock on fear learning which may also extend to certain measures of alcohol drinking (Quinn et al., unpublished data). Thus, the infant trauma model has the potential to uncover mechanisms driving sex- and stress-dependent vulnerabilities to alcohol drinking and the interactions between them.

As noted above for alcohol intake, important future work will also be needed to examine the potential relationship between estrous cycle and anxiety-like behaviors (and their interaction with drinking). For example, in premenstrual dysmorphic disorder, certain phases of the menstrual cycle can produce significant depression and anxiety symptoms, which is most commonly treated with chronic SSRI therapy, similar to other disorders of anxiety and depression [215]. Studies of the estrous cycle and anxiety-like behavior in rodents have both consistent patterns but also mixed findings. For example, several studies find reduced anxiety-like behavior during proestrus [74,105,106], while shock-licking behavior can be unrelated to estrous cycle phases [201]. Thus, understanding how anxiety/addiction are impacted by estrous cycle phases will be especially important for a nuanced and complete understanding of female anxiety and comorbid conditions.

## 7. Conclusions

Rodent models of binge and compulsion-like alcohol drinking have revealed a number of recent findings regarding sex differences in behavior and underlying mechanisms. Female vulnerability to alcohol is routinely seen in these models and under various conditions including operant, home cage self-administration, and conditioned place preference paradigms. Interestingly, some models may be able to parse out differences in specific aspects of alcohol drinking better than others. Thus, when designing and implementing experiments focused on investigating sex differences, it is imperative to consider how the chosen paradigm and variables (including alcohol concentration) can influence alcohol drinking behaviors. Another important consideration when studying sex differences is the influence of gonadal hormones, including cyclical variations in hormones due to the estrous cycle in females. Ovarian hormones such as estrogen play an important role in driving higher levels of consumption in female animals, although daily fluctuations due to estrous appear to be less consequential. Future studies should continue to explore the mechanisms by which estrogens enhance alcohol drinking, both within and outside of the mesolimbic dopamine system. Further exploration of the contributions of other gonadal hormones, such as progesterone and testosterone, as well as sex chromosomes is also warranted.

Our recent work has coalesced around the role of the NAcb in binge and compulsion-like alcohol drinking, and we find compelling evidence for the involvement of multiple subregions (i.e., core and shell), neuronal subtypes (i.e., D_1_- and D_2_-expressing medium spiny neurons), neurotransmitters (e.g., glutamate, orexin, and dopamine), and afferent projections to this region (e.g., mPFC and insula). Within these mechanisms, both sex-generalized and sex-specific effects have emerged. For example, both dopamine and orexin receptor antagonism (in the core vs. shell, respectively) reduced binge-alcohol consumption in male but not female mice, while inhibiting CP-AMPA receptors in the NAcb shell affected both sexes. Future work should aim to uncover additional mechanisms driving binge and compulsion-like alcohol drinking that are common vs. unique to males and females, and investigate the role of ovarian hormones and/or sex chromosomes in driving any observed sex differences. We also note that differences in stress processing and anxiety between males and females may contribute to differential alcohol drinking behaviors. Mechanistic explorations of how sex, stress, and the interactions between them alter neural circuits responsible for binge and compulsion-like alcohol drinking are likely to bring additional insights in the future.

Overall, sex differences in mental health conditions are observed clinically in humans but are not well understood mechanistically. Continuing to explore potential biological substrates in rodents to account for these sex differences is likely to make critical contributions for translation into human treatments. Additionally, reconsidering the validity and nuance through which we measure addiction and anxiety-like behaviors in rodent models is of increasing importance to ensure appropriate interpretations of results. Finally, as pathologic alcohol intake continues to rise in human females, it is important to examine the relationships between alcohol drinking, sex, and anxiety.

## Data Availability

Not applicable.
